# Proximal Femur Megaprostheses in Orthopedic Oncology: Evaluation of a Standardized Post-operative Rehabilitation Protocol

**DOI:** 10.1007/s43465-023-01092-1

**Published:** 2024-02-08

**Authors:** Lorenzo Andreani, Edoardo Ipponi, Federico Falcinelli, Martina Cordoni, Elena Bechini, Lorenzo Vannucci, Antonio D’Arienzo, Rodolfo Capanna

**Affiliations:** 1 Department of Orthopedics and Trauma Surgery, University of Pisa, Via Paradisa 2, 56124 Pisa, Italy; 2 Physiotherapy and Rehabilitation Unit, University of Pisa, Via Paradisa 2, 56124 Pisa, Italy

**Keywords:** Megaprosthesis, Proximal femur, Resection, Rehabilitation, Oncology, Bone tumor

## Abstract

**Background:**

Reconstructions of the proximal femur after massive resections represent one of the main challenges in orthopedic oncology. Among the possible treatments, megaprostheses represent one of the most used and reliable reconstructive approaches. Although literature about their outcomes has flourished through the last decades, a consensus rehabilitative treatment is still far from being established.

**Materials and methods:**

We evaluated the functional results of all our oncologic cases treated between 2016 and 2022 that could follow our standardized post-operative rehabilitative approach, consisting in progressive hip mobilization and early weight-bearing.

**Results:**

Twenty-two cases were included in our study. On average, their hospitalization lasted 15.1 days. The seated position was achieved on average within 3.7 days after surgery, the standing position reached 5.4 after surgery, while assisted deambulation was started 6.4 days after surgery. After a mean post-operative follow-up of 44.0 months, our patients’ mean MSTS score was 23.2 (10–30). Our data suggested a statistically significant inverse linear correlation between post-operative functionality and patients’ age, resection length, and the start of deambulation.

**Conclusions:**

A correct rehabilitation, focused on early mobilization and progressive weight-bearing, is crucial to maximize patients’ post-operative functional outcomes.

## Introduction

Megaprostheses are implantable devices designed to reproduce form and function of large bone segments, alongside with a variable share of their nearby soft tissues. Years of research and innovations shaped these prostheses with the aim to restore the functionality of patients who suffered massive bone and soft tissue losses, such as the ones that result from radical resections of bone tumors [1].

The history of endoprosthetic reconstruction in orthopedic oncology has its roots at the dawn between the 4th and the 5th decade of the twentieth century. In 1943, Moore and Bohlman first reported the use of a proximal femur prosthesis in a case with a giant cell tumor of bone [2]. In 1949, Seddon and Scales used a prosthetic implant to replace the proximal two-thirds of the femur in a case with fibrous dysplasia [3]. Since 1970, the introduction of multimodal therapies which improved patients’ survival, in association with the evolution of implants’ materials and manufacturing technologies, led to an ever-growing role for limb sparing surgery [4, 5]. Titanium alloys soon became the materials of choice for prosthetic stems of monoblock and modular megaprostheses, due to an elasticity similar to that of femoral bone [1]. The prosthetic fixation to the femoral shaft has been a challenge for long, due to the high junctional forces. In some of the first megaprostheses of proximal femur, intramedullary stems were furtherly stabilized with flanges that fit over the cortex [6]. Stem cementification has been largely used since the 1960s to secure the bone–prosthesis interface [7], while press-fit implants were a more recent innovation, targeted to achieve a better integration and minimize the risk of aseptic loosening that burdened cemented prostheses [8].

**Fig. 1:**
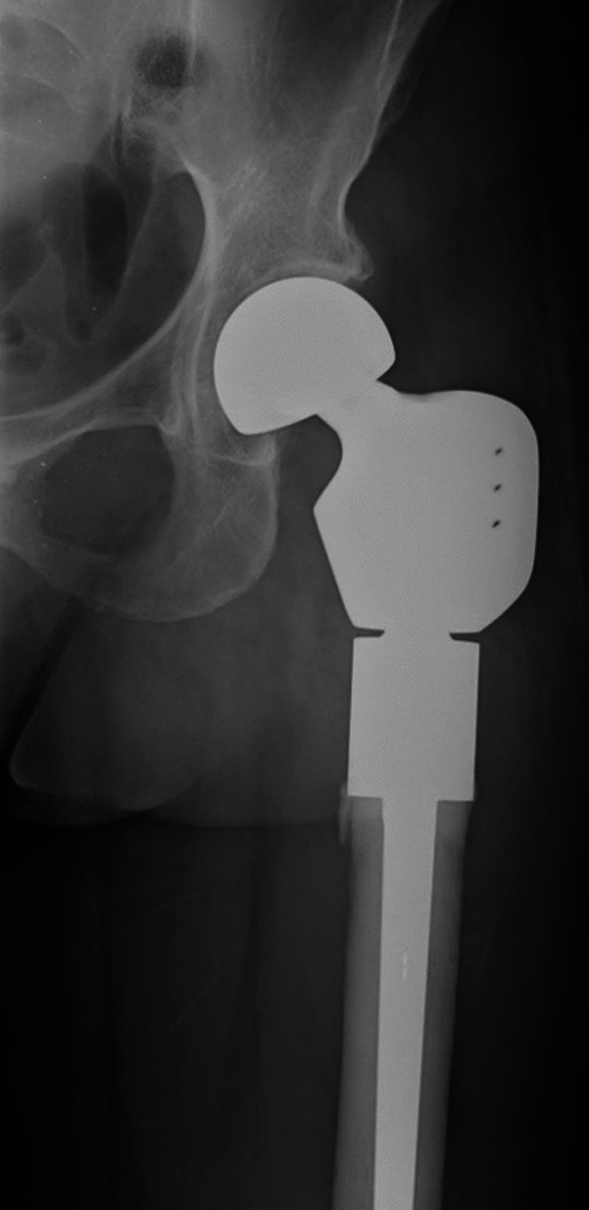
Post-operative X-Ray of a megaprosthesis of the proximal femur. Sites for the reattachment of the gluteal insertion are present in the trochanteric region of the implant

Despite the evolution of megaprostheses’ designs and materials, cases that received massive resections of their proximal femur are still burdened by the loss of soft tissue attachments. Cases that require the sacrifice or the detachment of the abductor muscles can develop a Trendelenburg gait, a painful limp, and have an increased risk of post-operative dislocation. Without the iliopsoas, hip flexion strength would be diminished. The detachment of the gluteus maximus insertion generally comprises leg extension and the ability to rise from a chair [1]. Although modern designs provide proximal femur megaprostheses with multiple sites for tendon reattachment (Fig. 1), the loss in muscular strength and soft tissue coverage inevitably cause reductions in hip strength and stability [9].

The maintenance of good muscular tone and tropism in the months and years that follow surgery might therefore play a pivotal role both in preventing implant dislocations and maximizing the functional performances of the implant.

Although literature flourished of notes about surgical techniques in cases with massive proximal femur resection and megaprosthetic reconstructions and much has been reported in terms of implants’ complications and post-operative performances, less attention has been paid to the rehabilitation protocols necessary to restore patients’ functionality. The paucity of evidence on post-operative physical treatments for cases that received a proximal femur megaprosthetic implant is also testified by the absence of consensus rehabilitation protocols.

In this study, we report our experience with our latest standardized rehabilitation program, evaluating the results we had on a mid-to-long-term scenario.

## Materials and methods

This single-center retrospective study was performed in accordance with the ethical standards laid down in the 1964 Declaration of Helsinki and its later amendments.

Our study consisted of a review of all the oncologic cases that have been treated in our institution with massive bone resection of the proximal femur and megaprosthetic reconstruction between June 2016 and June 2022.

Inclusion criteria were (I) a massive bone resection followed by the implant of a modular proximal femur megaprosthesis, (II) a certain diagnosis of primary or secondary bone tumor, and (III) the use of our standard rehabilitation protocol during and after patients’ hospitalization.

Exclusion criteria were (1) pre-operative neurological deficits or other systemic diseases that could have impeded the execution of a proper rehabilitation, (2) the intra-operative sacrifice of major motor nerve trunks in order to achieve wide resection margins, (3) the occurrence of post-operative mechanical failures or local recurrences that required further surgical interventions and thereby compromised patients’ post-operative intercourse, and (4) a follow-up shorter than 12 months.

For each patient, we recorded general data and their histological diagnosis. Pre-operative X-rays, CT scans, and MRI images were used in order to orient the pre-operative diagnostic process and to assess cases’ classification according to the Enneking classification for bone tumors. The same images were also used to aid surgical planning.

**Fig. 2:**
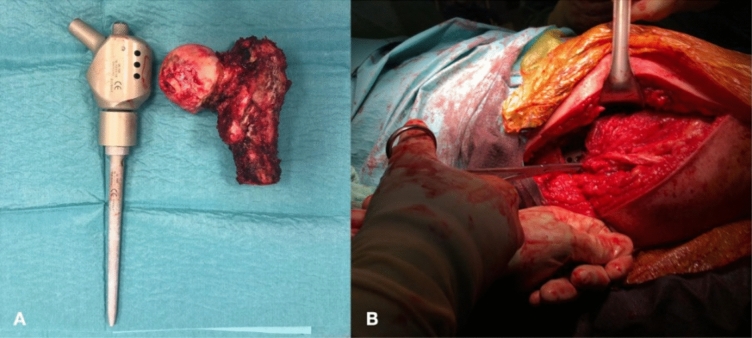
Intra-operative images of a proximal femur resection and megaprosthetic replacement. The proximal femur is resected and replaced with a prosthetic implant with comparable size and shape (**A**). The implant is then assembled and set in place; an adequate coverage of the quadriceps and gluteal region are necessary in order to achieve good post-operative stability and mobility (**B**)

Surgeries were all performed by the two expert surgeons RC and LA. Intra-operatively, we recorded the length of the resected bone segment. The megaprosthetic implant of choice was the Proximal Femur Megasystem C (Waldemar LINK^®^ GmbH & Co. KG, Hamburg, Germany). Eventual intra-operative complications were reported (Fig. 2).

Patients suffering from primary bone tumors received uncemented implants, whereas cemented stems were used for patients with metastatic diseases.

A total or subtotal resection of the hip joint capsule was performed in each case to achieve wide resection margins. While the design of our prosthesis did not allow the reinsertion of resected abductors into the prosthetic body, gluteal muscles, the other adductors, and the quadriceps could be anchored to the implant, playing a role as internal stabilizers. In the weeks and months that follow the surgical intervention, a fibrous cap would form to connect the proximal end of the prosthesis and the acetabular region, thereby mimicking a new articular capsule. No surgical mesh nor other local augments were used in our population. In the first weeks after surgery, an abduction hip brace was used to provide further external stabilization and minimize the risk of dislocation, while the fibrous pseudocapsule was still forming. In this scenario, the range of motion of the hinged brace was gradually increased to provide stability and respect the forming pseudocapsule while avoiding excessive hip rigidity [10]. A progressive weight-bearing was programmed to allow a gradual return to their activity for gluteal muscles and quadriceps, now sutured on the implant [10, 11].

All the cases included in our study carried out their post-operative rehabilitation according to our standardized protocol, described in detail in Table 1. The rehabilitation process of each case was shaped after our standardized protocol, although a certain grade of customization was allowed for each case. The extent of capsule remnants, when present, as well as the use of cemented or uncemented stems did not determine any change in our protocol. Cases received daily physical therapy under the supervision of our physiotherapy and rehabilitation unit.

**Table 1:** Rehabilitation Stages: Proximal femoral replacement with megaprosthesis

	1st week after surgery	2nd week after surgery	3rd-4th week after surgery	After the 1st month after surgery
Hip brace (degrees)	0–30°	0–30°	0–60°	0–60° (up to 0–90° in selected cases)
Hip brace (time)	Full-time	Full-time	Full-time	Full time for at least 2 months and progressive removal
Weight-bearing on operated leg	Toe-touch	Partial	Progressive partial	Full
Restoration and maintenance of muscle tone and tropism	+	+	+ +	+ + +
Postural passages	+ + +	+ + +	+ +	+
Gain re-education	+ + +	+ + +	+ + +	+ +
Stair climbing re-education	/	+	+ +	+ + +
Proprioceptive exercises	+	+	+	+ + +
Education about the hygienic and behavioral rules	+ + +	+ + +	+ + +	+ + +

The post-operative follow-up consisted of serial office visits, clinical evaluations, and X-rays images in order to assess clinical and radiological outcomes of surgical treatment. Each post-operative complication (Grade III or higher according to the Clavien—Dindo Classification) that did not represent an exclusion criterion has been reported. Complications were divided according to the Henderson failure mode. Each patient’s post-operative functional status was evaluated according to the MSTS score.

### Statistics

Statistical analysis was performed using Stata SE 13 (StataCorp LLC). Statistical significance was set at 0.05 for all endpoints.

## Results

Twenty-two patients with a bone tumor of their proximal femur who received massive bone resection and the implant of a megaprosthetic implant met our inclusion criteria and were therefore included in our study. The mean age of our cases was 58.9 (20–85).

Among our twenty-two cases, nine suffered from primary bone tumors: four cases were diagnosed with a chondrosarcoma, two cases had giant cell bone tumors of the bone, one had an Ewing sarcoma, another case suffered from a synovial sarcoma, and the remaining patient had a chondroblastoma associated with a secondary aneurysmal bone cyst. The remaining thirteen cases were suffering from metastatic lesions (twelve cases) or localizations of multiple myeloma (one case).

On average, the resected segment of the proximal femur had a length of 13.6 cm (9–25). None of our cases suffered from major intra-operative complications.

Among our cases, the mean hospital stay was 15.1 days (9–30). In these days, each case received active rehabilitative training by our physiotherapists. The seated position was achieved on average within 3.7 days after surgery (1–7). Our patients could reach the standing position on average 5.4 (2–9) after surgery, while assisted deambulation could be started within an average of 6.4 (4–9) days after surgery. Crutches were the first walking aids for 45.4% (10) of our cases, while the remaining 54.5% (12) resorted to walking frames.

Our patients’ mean post-operative follow-up was 44.0 (12–82) months.

Three of our cases suffered from major post-operative complications that did not represent exclusion criteria. One case had a dislocation of his prosthetic implant 6 months after surgery and was successfully treated with external traction under pharmacological sedation. Another patient had a seroma. The seroma was diagnosed in the weeks that came after surgery and was successfully treated with an ultrasound-guided percutaneous drainage. One case had a superficial wound infection, diagnosed within one month after surgery and successfully treated with antibiotic treatment and negative pressure wound therapy (NPWT).

At their latest follow-up, our patients’ mean MSTS score was 23.2 (10–30). The mean value for cases who were diagnosed with primary bone tumors was 25.4 (14–30), whereas the average value for cases with metastatic lesions was 21.4 (10–26).

A schematic resume of our population is reported in Table 2.

**Table 2:** A summary of the outcomes in our casuistry

Diagnosis	4 Chondrosarcoma 1 Synovial Sarcoma 1 Ewing Sarcoma 2 Giant Cell Tumor of the Bone 1 Chondroblastoma + ABC 1 Multiple Myeloma 12 Metastatic Carcinoma
Resection length (cm)	13.6 (9–25)
Hospitalization (days)	15.1 (9–30)
Seated position (day)	3.7 (1–7)
Standing position (day)	5.4 (2–9)
Deambulation (day)	6.4 (4–9)
Follow-up (months)	44 (12–82)
Post-op MSTS (/30)	23.2 (10–30)
Post-op complications*	

*POST-OP* Post-Operative. *ABC* Aneurysmal Bone Cyst

^*^Excluding those complications that represented exclusion criteria and issues of grade I or II according to the Clavien–Dindo classification

According to a Pearson correlation test, there was an inverse linear correlation between patients’ age and their post-operative functionality, as younger cases had significantly better MSTS scores at their latest follow-up (*r* = − 0.518; *p* = 0.013). Another Pearson correlation test highlighted a significant correlation also between the length of the resected bone and patients’ functional outcomes, since the larger the resections were, the worse were the post-operative MSTS scores (*r* = − 0.488; *p* = 0.021). Furthermore, a similar test testified that the sooner cases could start deambulation, the better their final functional outcomes came to be in terms of MSTS scores (*r* = − 0.480; *p* = 0.024).

Furthermore, a one-tailed T-student test suggested that cases with primary bone tumors without metastatic lesions at the moment of surgery had significantly better post-operative MSTS scores compared to the ones of those who had metastatic lesions (*t* = 1.728; *p* = 0.049).

## Discussion

Massive bone resections of the proximal femur and the subsequent reconstruction represent one of the main challenges in orthopedic oncology. Unlike common arthroplasty, where surgeons are called to replace only the surfaces of a damaged articulation, the implant of a megaprosthesis also allows the replacement of the trochanteric region and a variable share of the femoral shaft [12–15].

The functional results that follow the implant of these prosthesis can be influenced by several factors including patients’ pre-operative general conditions and local factors, including resection length and the sacrifice of the nearby soft tissues that are necessary to provide stability and allow hip movements [13–18].

Among patients’ generalities, age can represent a crucial factor to predict the effectiveness of proximal femur reconstructive surgery in terms of functional outcomes [19]. In fact, patients’ age could theoretically influence the quality of patients’ bones and the healing capacity of the surrounding soft tissues [20, 21]. Furthermore, older cases are more frequently affected by concomitant diseases that could delay local healing, increase the risk of local complications, and retard the rehabilitation protocol. Our cohort highlighted a statistically significant correlation between patients’ age and post-operative functionality. Younger cases had significantly better functional outcomes compared to the older ones. Considering that mechanical failures and local recurrences represented exclusion criteria for our study, this correlation seems to be mainly attributable to the quality of treated bones and soft tissues and patients’ global conditions.

From a local point of view, literature suggests that the extension of the tumor and the width of the consequential sacrifice of bone stock, ligaments, muscles and tendons in the hip, gluteal and thigh region might be a pivotal factor to influence the stability and mobility of the megaprosthesis [13–19]. In fact, the reattachment of what remains of the soft tissues that surround the prosthesis is commonly considered as a key requirement to determine the functional success of the implant [22, 23]. Although modern devices are designed with anchoring points in strategic positions to allow a better reinsertion of resected muscles and tendons, the pursuit of wide margins of resection during the demolition phase can result in a lack of adequate muscular stock. Thereby, wider femur resections and more extended sacrifices of the nearby soft tissues could be associated with lower implant stability and less muscular strength [11, 13–19, 22]. Our cases support this line of reasoning. In our casuistry there was a statistically significant correlation between the length of the resected proximal femur and the post-operative functional impairment, as cases with larger resections and longer implants had significantly lower functional outcomes, assessed using the MSTS score.

Once surgery is over, a proper rehabilitation is necessary in order to maximize the effectiveness of the megaprosthesis. Although the role of rehabilitation in modern arthroplasty is unquestioned, to this date, literature lacks detailed rehabilitative protocols for cases who underwent massive resection of their proximal femur and megaprosthetic reconstruction. The aim of our study was to propose our standardized rehabilitation protocol as a guide-line for those who are new to this surgical technique and for those who are using different approaches. In particular, our rehabilitation protocol, described in detail in Table 1, is focused on a progressive return to weight-bearing on the treated lower limb in association with an early active and passive mobilization finalized to a correct re-education of the patient and to the restoration and the maintenance of muscle tone and tropism. Within the first two months after surgery, an articulated hip brace locked at 0–60 degrees is useful in order to protect the joint and to guide hip movements, allowing adequate healing of the muscle components detached from the resected bone portion and reinserted on the megaprosthesis [23].

Our experience suggests that proper physical treatments are mandatory to allow the maintenance of acceptable muscular tone and strength which are necessary for the correct mobility and stability of the replaced hip. This idea has been also reinforced by the finding that, in our population, the earlier cases that could start walking, the better their functional outcomes came to be according to the MSTS scoring system. This evidence suggests that a correct mobilization and avoiding delays in patients’ walking permission could be crucial to maximize the performances of cases treated with proximal femur resection and reconstruction with megaprosthetic implants.

We acknowledge our study is not free of limitations. One of them is represented by the retrospective nature of our study, which did not allow the complete standardization of the post-operative follow‐up procedures for each patient. Another limitation is represented by the short size of our cohort. The rarity of these tumors and the limited timespan of investigation did not allow us to operate on a wider population, which partially limited the statistical significance of some of the data associations we wanted to investigate at the beginning of our research. Both these issues could be overcome in the next future performing similar evaluations on a prospective basis and on wider populations.

Beyond these limitations, our outcomes confirm that factors such as age and resection length are associated with patients’ post-operative functionality. Rehabilitation, focused on early mobilization and progressive weight-bearing, represents another crucial factor to allow patients to achieve their best post-operative functional outcomes. Finally, better performances of the prosthetic implant and the surrounding soft tissues could allow patients to return to their activities of daily living, promoting their quality of life.

## Conclusion

Megaprostheses are among the most used reconstructive approaches in cases with bone tumors. The resection of the proximal femur and its replacement with a prosthetic implant represents one of the main challenges in orthopedic oncology. In parallel with the surgical technique, a proper and accurate rehabilitation protocol is necessary in order to maximize patients’ post-operative functional outcomes, thereby increasing their quality of life.
